# Antiviral Efficacy of Molnupiravir for COVID-19 Treatment

**DOI:** 10.3390/v14040763

**Published:** 2022-04-06

**Authors:** Yuan Bai, Mingwang Shen, Lei Zhang

**Affiliations:** 1 WHO Collaborating Centre for Infectious Disease Epidemiology and Control, School of Public Health, Li Ka Shing Faculty of Medicine, The University of Hong Kong, Hong Kong, China; yb424@hku.hk; 2 Laboratory of Data Discovery for Health, Hong Kong Science and Technology Park, Hong Kong, China; 3 China-Australia Joint Research Center for Infectious Diseases, School of Public Health, Xi’an Jiaotong University Health Science Center, Xi’an 710061, China; lei.zhang1@xjtu.edu.cn; 4 Artificial Intelligence and Modelling in Epidemiology Program, Melbourne Sexual Health Centre, Alfred Health, Melbourne, VIC 3004, Australia; 5 Central Clinical School, Faculty of Medicine, Monash University, Melbourne, VIC 3800, Australia; 6 Department of Epidemiology and Biostatistics, College of Public Health, Zhengzhou University, Zhengzhou 450001, China

**Keywords:** COVID-19, SARS-CoV-2, Molnupiravir, antiviral efficacy

## Abstract

The ongoing global pandemic of COVID-19 poses unprecedented public health risks for governments and societies around the world, which have been exacerbated by the emergence of SARS-CoV-2 variants. Pharmaceutical interventions with high antiviral efficacy are expected to delay and contain the COVID-19 pandemic. Molnupiravir, as an oral antiviral prodrug, is active against SARS-CoV-2 and is now (23 February 2022) one of the seven widely-used coronavirus treatments. To estimate its antiviral efficacy of Molnupiravir, we built a granular mathematical within-host model. We find that the antiviral efficacy of Molnupiravir to stop the growth of the virus is 0.56 (95% CI: 0.49, 0.64), which could inhibit 56% of the replication of infected cells per day. There has been good progress in developing high-efficacy antiviral drugs that rapidly reduce viral load and may also reduce the infectiousness of treated cases if administered as early as possible.

## 1. Introduction

The ongoing global pandemic of COVID-19 has offered the world a crash course in modern epidemiology [1], motivating countries to launch economic recovery programs to mitigate unemployment [2]. Coupled with mass vaccination, COVID-19 antivirals are needed to limit virus spread and change the course of the pandemic. Molnupiravir is a small-molecule antiviral prodrug against SARS-CoV-2 virus [3]. Now (23 February 2022) it is one of the seven widely-used coronavirus treatments [4]. Together with Paxlovid, it is one of two promising antiviral pills developed in 2021 which may change the course of the pandemic [5]. The full data concerning viral load dynamics of Molnupiravir was released on the 10 February 2022 [3].

Although mathematical models of COVID-19 transmission at the population level have been useful in controlling the spread of the virus, understanding of the dynamics of infection within an infected cell is still lacking. Therefore, it is difficult to study the viral dynamics inside an infected cell in detail and the effects of antiviral treatments on the infection dynamics within an infected cell [6]. Coronaviruses have the largest genomes and the most unique viral life cycle of any single-stranded RNA virus, with the result that current intracellular infection models cannot be directly applied [6]. Several key epidemiological metrics (e.g., virus infection rate, virus replication rate, virus clearance rate, and cell death rate) are needed to characterize the within-host dynamics of the SARS-CoV-2 virus, together with the antiviral efficacy. Robust estimates of their distributions are essential for determining the viral life cycle. We conducted a modeling study on within-host dynamics of the SARS-CoV-2 virus to assess the antiviral efficacy of Molnupiravir to inhibit the replication of infected cells.

## 2. Methods

**Figure 1:**
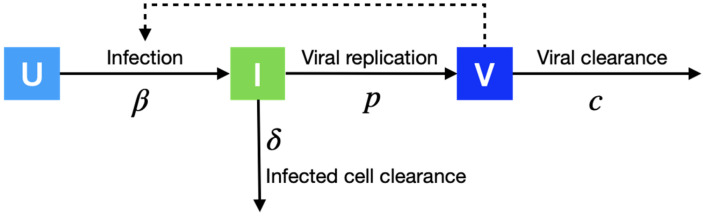
Within-host modeling. We model the replication dynamics of SARS-CoV-2 viruses within an infected individual. Uninfected cells (*U*) progress to infectious cells (*I*) with infection rate β, and finally release viruses (*V*) with replication rate *p*.

We model the effect of antiviral treatment on the within-host proliferation of cells infected by the SARS-CoV-2 virus using the target cell model (Figure 1). Let U be the number of susceptible cells at risk, *I* the number of infected cells, and *V* the number of active viruses, respectively. We model the replication dynamics of SARS-CoV-2 viruses within each infected individual described by the following ordinary differential equations:

$$\frac{dU}{dt}=-\beta UV$$

$$\frac{dI}{dt}=\beta UV-\delta I$$

$$\frac{dV}{dt}=\left(1-\epsilon \right)pI-cV$$

where the interaction between susceptible cells and active viruses leads to the infection of susceptible cells at rate β, the infected cells die at rate δ, the viruses replicate at rate *p*, the antiviral drug inhibits the replication of infected cells at rate ϵ (i.e., antiviral efficacy), and the active viruses die at rate *c*, respectively. We fix the initial number of viruses (*V*_0_) as 1/30 copies/mL [7] and the initial number of target cells (U_0_) as 10^7^ [8].

For the viral load data, Angélica et al. [3] identified 1433 COVID-19 infected, non-hospitalized, unvaccinated adults, of which 716 received the Molnupiravir treatment. They published their viral load values (log_10_ copies/mL). There are 5 days on average from the COVID-19 exposure to symptom onset [9]. There are 3 days on average from COVID-19 symptom onset to randomization in study adults [3], resulting in 8 days from the exposure to the treatment. 

To evaluate the impact of antiviral therapy on the transmission dynamics of COVID-19, we model the relationship between the viral load and infectiousness of an individual. We adopt a logarithmic relationship between the transmission rate and viral titer (which requires fewer parameters than a sigmoidal function). Specifically, with viral dynamics, the transmission rate, denoted by Γ, will change over time post-infection. For an individual infected at *T*, at time *t*, it can be estimated as:

$$\Gamma \left(t\right)=\phi log\left(V\left[t\right]\right)$$

where ϕ is the population baseline transmission rate estimated from the real data, and *V*(*t*) is the viral load of this individual infected at time t post-infection, which is set to be zero when less than the virus detection threshold of 100 [10]. We evaluate the infectiousness of an infected individual by summing Γ(*t*) over time.

## 3. Parameter Calibration of Within-Host Modeling

We used a nonlinear model to fit the within-host model (Figure 1) to the viral load data in the clinical data [3]. The nonlinear model included both a fixed effect, which is constant in both the placebo and Molnupiravir groups, and a random effect, which is different between the placebo and Molnupiravir groups, in each fitted parameter. The two effects were estimated using the stochastic approximation expectation–maximization algorithm and empirical Bayes method, respectively [11]. We calibrate the cell infection rate in 10^−6^ days^−1^ (β), infected cell death rate in days^−1^ (δ), virus production rate in Copies/mL in days^−1^ (*p*), virus death rate in days^−1^ (*c*), which are assumed to be the same in the two study groups, and antiviral efficacy (ϵ), which is assumed to be zero in the placebo group and fitted value in the Molnupiravir group, using the Metropolis–Hastings algorithm. The parameter calibration was performed using MONOLIX 2021R1 [11]. 

## 4. Results

We model the within-host dynamics of infected individuals who have an initial virus load which increases via replication and decreases via the immune response and antiviral treatment. We estimated the antiviral efficacy with which Molnupiravir inhibits viral replication by fitting the model to a recent clinical trial [3] that measured the viral dynamics of 1433 SARS-CoV-2-infected adults after treatment with Molnupiravir (549 patients) or a placebo (555 patients). Within 3 days of initiating Molnupiravir oral treatment, the virus load had decreased by an estimated 88%, compared with an expected reduction in untreated cases of 84%. 

**Figure 2:**
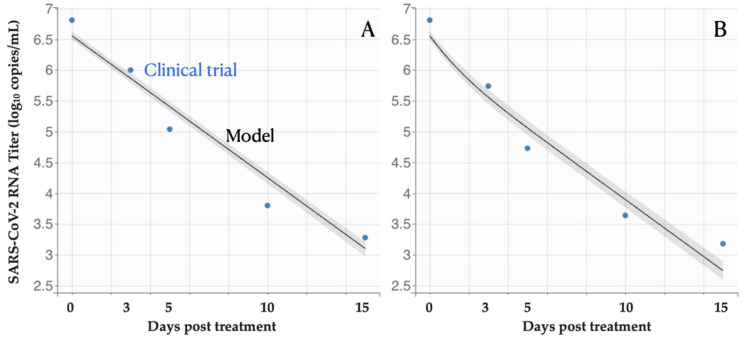
Viral load following Molnupiravir treatment. The estimated means and 95% CI of virus titer from the fitted within-host model track the mean values of empirical observations (blue circles) among patients treated with (**A**) placebo, or (**B**) Molnupiravir. Day zero corresponds to the beginning of the treatment.

Our in-host model produces viral titer estimates similar to the mean clinical data (Figure 2). Using a nonlinear mixed-effects model, we estimate the antiviral efficacy of 0.56 (95% CI: 0.49, 0.64) (Table 1), indicating that the Molnupiravir treatment could inhibit 56% of replication of infected cells per day. Furthermore, the cell infection rate, the infected cell death rate, the virus production rate, and the virus death rate are estimated to be 2.8 (95% CI: 2.16, 3.69) 10^−6^ days^−1^, 0.53 (95% CI: 0.52, 0.54) days^−1^, 10.96 (95% CI: 9.65, 12.40) Copies/mL in days^−1^, and 1.33 (95% CI: 1.26, 1.41) days^−1^, respectively. Our estimates are close to the values reported in other studies.

We fitted the within-host model to the mean viral load by fixing the initial number of viruses (*V*_0_) as 1/30 copies/mL following the COVID-19 viral load study in ref. [7], and the corresponding parameter fitting values are shown in Table 1. While some researchers would like to assume *V*_0_ as other values without clear explanations, for example, 4.5 copies/mL in ref. [15] and 357 [12], we conducted a sensitivity analysis by varying *V*_0_ from 1 to 1000 and re-estimated those parameters (Table 2). We found the Cell infection rate in 10^−6^ days^−1^ (β) varies obviously between scenarios, whereas these parameter estimations are robust, especially for the antiviral efficacy with a small variation over scenarios.

Using these inferred parameter values, we estimate the infectiousness of an infected individual will be reduced by 9%, 8% and 7% if the infected individual receives the Molnupiravir treatment on day 2, 5 and 8 post-infection, respectively. If we assume the effective reproduction number of COVID-19 is 5 and all infected people take the Molnupiravir treatment on day 2, 5 and 8 post-infection, the reduced infectiousness will reduce the effective reproduction number to 4.57, 4.62, and 4.66, respectively.

If the antiviral efficacy increases to 1, the infectiousness will reduce by 62%, 48% and 38% when receiving the Molnupiravir treatment on day 2, 5 and 8 post-infection, respectively. The effective reproduction number of COVID-19 will then reduce from 5 to 1.92, 2.61, and 3.08, respectively.

## 5. Discussion

Early Molnupiravir treatment for COVID-19 significantly reduced SARS-CoV-2 log viral load by 1.9% in infected participants who were still positive for infectious virus within 3 days of initialization of Molnupiravir treatment [16]. Informed by the recent clinical study published on the 10 February 2022 [3], we estimated the antiviral efficacy of Molnupiravir is 0.56 (95% CI: 0.49, 0.64), which could inhibit 56% of replication of infected cells per day. Although Molnupiravir treatment has a significant efficacy with the result that the risk of hospitalization or death is reduced by half compared with a placebo, it has limited potential to inhibit virus replication.

Future epidemiological studies and clinical trials may allow us to resolve such limitations and capture two main complexities that are not yet included in our models. First, we did not consider that viral kinetics and treatment efficacy may vary substantially across age groups and risk groups, as other researchers have demonstrated [17,18]. We expect the inclusion of this variability will enhance intervention assessments and prioritization of medical services but not alter the results of this study qualitatively. Second, we are not yet modeling the development and spread of antiviral-resistant viruses, which can modify the effects of ramping up treatment rates at the population level.

Efforts to achieve a successful treatment have focused on repurposing current approved drugs for its use as antivirals [19]. Promising candidates included nucleoside analogs such as remdesivir (originally designed to treat Ebola and Hepatitis C) [20,21,22,23], favipiravir (originally designed to treat influenza) [24], and dexamethasone (an anti-inflammatory drug) [25]. As public health agencies worldwide have their patients treated by timing the ramp-up of its antiviral treatment coverage, estimates of antiviral efficacy would highlight the potential benefits of pharmaceutical action of antiviral drug development, especially as close to the nation’s full reopening as possible.

In conclusion, our results indicate the high antiviral efficacy of the antiviral treatment, which may avert substantial COVID-19 cases as this treatment scales up. The development of a Molnupiravir-like drug for SARS-CoV-2 cannot suppress the viral load within a few days, but shortens the contagious period significantly. As public health agencies around the globe struggle to determine when to implement potentially costly social distancing measures, these estimates highlight the potential benefits of high-efficacy antiviral drugs. Their application could finally isolate COVID-19 cases pharmaceutically rather than physically, and thereby disrupt chains of transmission. 

## Figures and Tables

**Table 1:** Within-host parameter estimates. We fitted the within-host model to the mean viral load dynamics of 1433 infected adults [3] by using nonlinear mixed-effects model method [11] to infer the parameters.

Parameter	Estimation	Similar Estimates
Cell infection rate in 10^−6^ days^−1^ (*β*)	2.8 (95% CI: 2.16, 3.69)	1.6 [12]
Infected cell death rate in days^−1^ (*δ*)	0.53 (95% CI: 0.52, 0.54)	0.65 [13]
Virus production rate in Copies/mL in days^−1^ (*p*)	10.96 (95% CI: 9.65, 12.40)	8.57 (95% CI: 5.01, 12.58) [14]
Virus death rate in days^−1^ (*c*)	1.33 (95% CI: 1.26, 1.41)	1.75 (95% CI: 0, 3.55) [6]
Antiviral efficacy (*ϵ*)	0.56 (95% CI: 0.49, 0.64)	Not appliable

**Table 2:** Sensitivity analysis of antiviral efficacy by varying initial viral load. We conducted the sensitivity analysis of parameter calibration by varying the initial number of viruses in copies/mL from 1 to 1000.

Parameter	Scenario V1	Scenario V2	Scenario V3	Scenario V4
Initial number of viruses in copies/mL (*V_0_*), assumed	1	10	100	1000
Cell infection rate in 10^−6^ days^−1^ (β), calibrated	1.74 (95% CI:1.27, 2.42)	0.87 (95% CI:0.73, 1.03)	1.36 (95% CI:0.94, 1.97)	1.15 (95% CI:0.74, 1.8)
Infected cell death rate in days^−1^ (δ), calibrated	0.54 (95% CI:0.53, 0.55)	0.53 (95% CI:0.52, 0.54)	0.53 (95% CI:0.52, 0.54)	0.53 (95% CI:0.52, 0.53)
Virus production rate in Copies/mL in days^−1^ (*p*), calibrated	12.01 (95% CI:9.97, 14.52)	10.55 (95% CI:8.38, 13.34)	11.29 (95% CI:9.93, 13.03)	12.26 (95% CI:10.88, 13.9)
Virus death rate in days^−1^ (*c*), calibrated	1.35 (95% CI:1.27, 1.43)	1.51 (95% CI:1.43, 1.6)	1.29 (95% CI:1.17, 1.43)	1.36 (95% CI:1.28, 1.45)
Antiviral efficacy (ϵ), calibrated	0.55 (95% CI:0.45, 0.65)	0.55 (95% CI:0.47, 0.63)	0.62 (95% CI:0.54, 0.69)	0.53 (95% CI:0.45, 0.62)

## Data Availability

All data are collected from open sources with detailed description in Methods section.
